# Differential Expression Analysis Utilizing Condition-Specific Metabolic Pathways

**DOI:** 10.3390/metabo13111127

**Published:** 2023-11-03

**Authors:** Gianluca Mattei, Zhuohui Gan, Matteo Ramazzotti, Bernhard O. Palsson, Daniel C. Zielinski

**Affiliations:** 1 Department of Experimental and Clinical Biomedical Sciences, University of Florence, 50121 Florence, Italy; gianluca.mattei@unifi.it (G.M.);; 2 School of Basic Medical Sciences, Wenzhou Medical University, Wenzhou 325035, China; zgan@eng.ucsd.edu; 3 Department of Bioengineering, University of California San Diego, La Jolla, CA 92093-0412, USA

**Keywords:** pathway analysis, metabolism, constraint-based modeling, expression analysis

## Abstract

Pathway analysis is ubiquitous in biological data analysis due to the ability to integrate small simultaneous changes in functionally related components. While pathways are often defined based on either manual curation or network topological properties, an attractive alternative is to generate pathways around specific functions, in which metabolism can be defined as the production and consumption of specific metabolites. In this work, we present an algorithm, termed MetPath, that calculates pathways for condition-specific production and consumption of specific metabolites. We demonstrate that these pathways have several useful properties. Pathways calculated in this manner (1) take into account the condition-specific metabolic role of a gene product, (2) are localized around defined metabolic functions, and (3) quantitatively weigh the importance of expression to a function based on the flux contribution of the gene product. We demonstrate how these pathways elucidate network interactions between genes across different growth conditions and between cell types. Furthermore, the calculated pathways compare favorably to manually curated pathways in predicting the expression correlation between genes. To facilitate the use of these pathways, we have generated a large compendium of pathways under different growth conditions for *E. coli*. The MetPath algorithm provides a useful tool for metabolic network-based statistical analyses of high-throughput data.

## 1. Introduction

High-throughput molecular data such as genome-scale gene expression measurements are ubiquitous; however, this abundance of data comes with the challenge of efficiently extracting biological knowledge from the data. It is largely this challenge that has catalyzed the rise of systems biology [1]. As cells are fundamentally composed of networks of interacting molecules, the central challenge in data analysis becomes understanding molecular interactions in the context of the underlying biochemical network [2,3]. As one of the primary tools of systems biology, biochemical pathway-based analysis is attractive for its ability to integrate signals from multiple functionally connected components, which can amplify the statistical power of coordinated changes in the system [4]. Within metabolism specifically, pathway analysis plays a particularly important role [4,5,6] as connections between enzymes have clear functional objectives in the conversion of molecules to energy, biomass, and other functional molecules. However, identifying pathway structures that are best used to interpret high-throughput data remains an active area of research [7].

Metabolic pathways have historically been manually defined based on an intuitive understanding of the function of particular sets of enzymes. Classical pathway examples such as glycolysis and the TCA cycle appear in textbooks and pathway databases and have well-accepted structure and content [5,8]. However, canonically defined pathways are not necessarily the most optimal pathways for analysis. For example, these pathways do not generally take into account the myriad variations in metabolism that exist across the phylogenetic tree [9], the condition-specific use of pathways and partial pathways, or the in vivo tight coupling of canonically separate pathways, such as the coupling of biosynthesis of a biomass precursor to the synthesis of required cofactors for a pathway [7]. As alternatives to manually defined pathways, a number of methods have been developed to algorithmically calculate pathways from a metabolic network structure directly [7]. These algorithmic methods often have a strict numerical objective, such as calculating extreme pathways according to a non-negative basis for the null space of the stoichiometric matrix of the metabolic network [10]. As these methods are rooted in the structure of organism-specific metabolic networks, calculated pathways have the benefit of accounting for organism-specific and condition-specific pathway variation. However, pathways defined based on purely numerical criteria still may not be the most practical or the most effective pathways to interpret high-throughput data, and their calculation on the genome scale can become computationally infeasible. Ideally, pathways would be defined in a way that is both functionally intuitive and accounts for organism- and condition-specific nuances.

The first challenge in designing a pathway-based data analysis is to obtain a functional definition of a pathway, which we propose can be defined as a set of genes working together to achieve a function. In a metabolic network, function can be defined succinctly in terms of the production and degradation of metabolites. Using these definitions, we can define a metabolic pathway as a sequential set of enzymes that is involved in the production or degradation of a metabolite under a defined metabolic flux state. This definition inherently assumes that the flux directions through the network are defined, but in fact, these are not fixed across growth conditions. A set of reactions may be involved in the consumption of a metabolite under one condition and the production of the metabolite under another condition, for example, the relationship between the reactions involving glycolysis and glucose under glycolytic compared to gluconeogenic conditions. Thus, the functional interpretation of changes in enzyme levels depends on the flux directions in the network. Furthermore, the relative importance of enzyme changes depends on the contribution of a pathway to a metabolic function. As an example, the pathways of glycolysis and glycogen synthesis both consume glucose. However, glycolysis operates at a rate greatly exceeding that of glycogen synthesis. Thus, a 10% increase in expression of glycolytic enzymes presumably would have a much greater impact on glucose turnover than a 10% decrease in glycogen synthesis. We can use this type of analysis to examine how gene expression changes in these pathways in order to identify coordinated expression shifts that serve specific metabolic functions in terms of increased or decreased capacity for the production or degradation of specific metabolites.

In this work, we developed a constraint-based modeling method, termed MetPath, to calculate condition-specific production and consumption pathways for the purpose of differential analysis of metabolic expression data. Using metabolic modeling, we calculated metabolic pathways for specific flux conditions by defining weighted, context-specific pathways in terms of metabolic functions, i.e., production/consumption of specific metabolites. We compared this method qualitatively and quantitatively to existing pathway databases, specifically the KEGG database, and found that the MetPath pathways show a higher intergene correlation within the same pathways across different conditions in *E. coli* K12 MG1655. We examined the performance of MetPath using two case studies in *E. coli*, looking at the tryptophan pathway during aerobic growth on glucose with and without tryptophan supplementation as well as glucose growth under the condition of aerobic–anaerobic shift. Finally, we looked at the ability of MetPath pathways to interpret cell-specific differences in gene expression, specifically examining neurotransmitter pathway expression in human neural cell subtypes based on single-cell transcriptomics data.

## 2. Materials and Methods

### 2.1. Overview of MetPath Pathway Calculation and Differential Gene Expression Analysis

**Figure 1:**
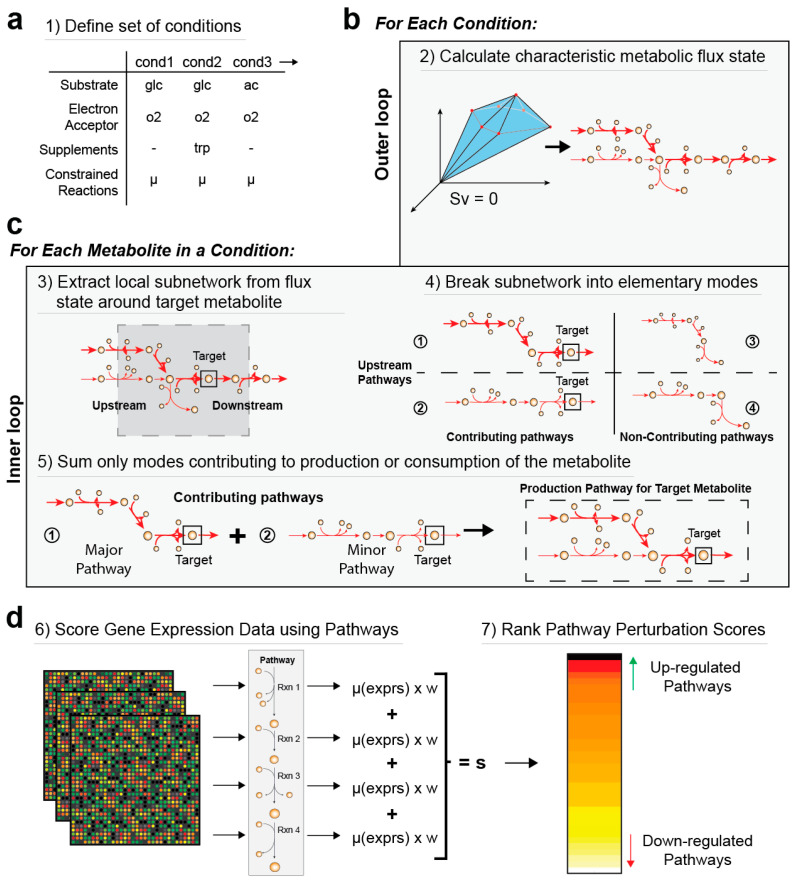
Overview of the MetPath algorithm. MetPath is a constraint-based modeling method to calculate pathways for the production and consumption of each metabolite in a network. (**a**) The algorithm begins by calculating a flux state for the condition of interest. (**b**) Then, for each metabolite, a subnetwork around the metabolite that is active based on the flux state for the condition is extracted. (**c**) This subnetwork is then broken down into production pathways and consumption pathways, weighted by their flux contribution, using elementary modes for each metabolite in the network. (**d**) To interpret differential gene expression data using MetPath pathways, a reaction score is calculated for each reaction in the pathway as the multiplication of differential gene expression for genes catalyzing the reaction based on the weighting of that reaction within the pathway. These reaction scores are summed and divided by the number of reactions to obtain a pathway score. A value of 1 indicates unchanged expression for the pathway, a value greater than 1 indicates an up-regulation of genes in the pathway, and a value less than 1 indicates a down-regulation of genes in the pathway. These scores are then ranked, and highly perturbed consumption and production pathways are identified.

We define the functions of a metabolic network as the production and degradation of metabolites (Figure 1). Utilizing this definition of metabolic function, we use constraint-based modeling to calculate the pathways that are involved in the production or degradation of each metabolite under a specified metabolic flux state. Finally, we assess, in the form of a perturbation score, how gene expression has changed in these pathways. This workflow identifies coordinated expression shifts that serve specific metabolic functions in terms of increased or decreased capacity for the production or degradation of specific metabolites. The source code can be found at github.com/SBRG/MetPath.

### 2.2. Calculation of a Condition-Specific Flux State

To calculate state-specific production and degradation pathways, we first calculate the estimated metabolic state. We solve the quadratic programming flux balance analysis problem of a metabolic model constrained by the estimated metabolite uptakes as follows:

$$\max Z=c^{T}v+vFv^{T}$$

Sv=0

$$v_{lb}<v<v_{ub}$$

where Z is the objective score; v is the vector of reaction fluxes; S is the matrix of reaction stoichiometries; v_lb_ and v_ub_ are the lower and upper bounds of the reaction fluxes, respectively; c is the linear objective vector, which is typically the biomass reaction but can be changed by the user; and F is a diagonal scaling matrix used as a secondary flux length objective and is set to an arbitrarily small value of 10^−6^. The flux state is calculated by minimizing the total length of the flux vector subjected to constraints, and represents the principle that a cell will try to achieve its metabolic function using as little enzyme expenditure as possible to minimize precursor costs. The purpose of this flux state estimation is to identify possible reaction directions and relative pathway flux values, given the established literature on aspects such as metabolite synthesis versus de novo uptake and relative energy production between glycolysis and beta-oxidation. These relative weightings and pathway directions add important information when calculating production and degradation pathways, as they lend context to the interpretation of a gene expression change related to the potential for production and degradation of different metabolites in the network.

### 2.3. Calculation of Production and Degradation Pathways for Each Metabolite

Then, using this estimated flux state, we calculate weighted production and degradation pathways for each metabolite as follows: First, reactions that carry flux in the estimated flux state are extracted as zero-flux reactions do not contribute to pathways under the selected condition. Then, a desired pathway length D is defined. For each metabolite, reactions that are within the distance D by means of a forward traversal (in the case of degradation) or reverse traversal (in the case of production) of the flux-carrying network are identified. For non-cofactor metabolites, cofactors are first removed from the network before traversal to prevent spurious connections. The production or degradation pathway subnetworks are then extracted and mass balanced by adding compensating input and output reactions for unbalanced metabolites. These subnetworks are then broken down into elementary modes using a published algorithm [11]. These elementary modes are mass-balanced pathways with weightings that, when summed, recapitulate the full flux distribution. Elementary mode pathways that contain the current metabolite are then extracted and summed to create a single weighted production or degradation pathway for the metabolite, representing the contribution of reactions within the distance D to the production or degradation of the metabolite in the estimated flux state. In practice, we found that distances D between 2 and 5 yielded similar performance in terms of intra-pathway expression correlation, with a distance of 1 yielding worse performance.2.4. Construction of Aggregate Pathway Perturbation Scores

To construct perturbed production and degradation scores for each metabolite, we first define reaction fold-change scores by averaging the fold change for all genes that are involved in the catalysis of each reaction as follows:

(1) $$reaction\_score=\frac{1}{n}\sum_{i=1}^{n}f_{i}$$

where n is the number of genes, and f is the fold change for each gene. We then define the production and degradation pathway perturbation scores for each metabolite by calculating a weighted average of the pathways with their corresponding reaction expression fold changes as follows:

(2) $$pathway\_score=\sum_{i=1}^{n}w_{i}\times {reaction\_score}_{i}$$

The reaction weightings w are assigned according to the reaction fluxes within each pathway and are normalized by the sum of those fluxes. These final production and degradation scores for each metabolite represent the expression change in reactions involved in the production and degradation of the metabolite, respectively, weighted by the degree of contribution of each reaction to the metabolite production/degradation in the estimated flux state.

### 2.4. Universal Database Generation

The universal database was constructed by deploying MetPath using common growing conditions for *E. coli* cultures. With this purpose, we simulated 64 different growing conditions. Starting from what we considered to be the standard growing conditions (minimal medium, 37 °C), we obtained 20 different cultures, each characterized by a supplement of a single amino acid (Supplementary Data File S1). In these cases, the lower bound of the specific amino acid was set to −0.5 to activate its uptake and the downstream reactions. For all these cases, flux distributions were calculated under both aerobic conditions and anaerobic conditions. We also simulated cultures with different carbon sources (glucose, lactate, galactose, mannose, acetate, fumarate, succinate, and glycolate). These cultures were matched with normoxia, hypoxia, and anoxia using nitrate as electron acceptors. The relative data used to constrain the models were taken from the literature [12]. For both amino acid-supplemented conditions and conditions with different carbon sources, before setting the ATP production as the objective function, the minimum biomass was set to the maximum of the model capacities.

### 2.5. KEGG Comparison

**Figure 2:**
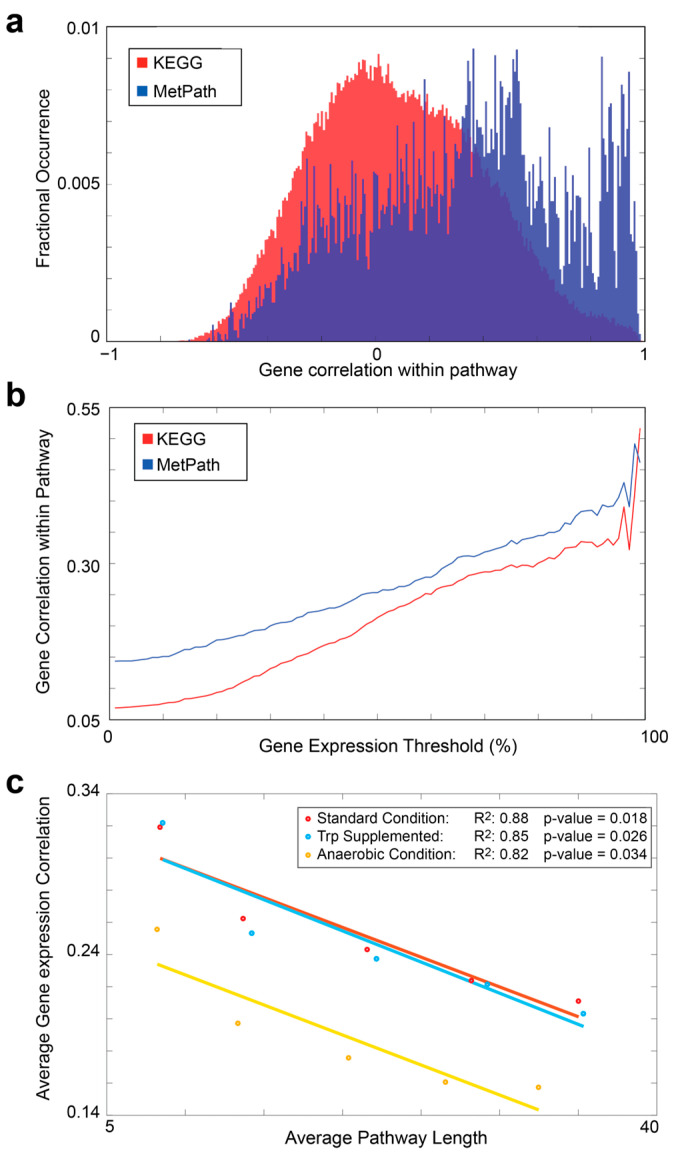
Comparison of MetPath and KEGG pathways in predicting gene expression correlation. (**a**) Histograms of the correlation of gene expression within the pathways for KEGG (red) and the MetPath (blue) pathways. Expression data were obtained from 213 samples under various conditions in *E. coli* K12 MG1655. (**b**) Gene correlation within the pathways as a function of the expression level of each gene. Highly expressed genes show greater correlation with each other. (**c**) Correlation of gene expression for genes within the pathways when compared at different pathway lengths. A clear negative correlation is observed, demonstrating that the correlation between genes diminishes rapidly with an increase in network distance between the genes.

To perform the comparison with data from the KEGG pathway database, we used an *E. coli* K-12 MG1655 array expression database obtained from 213 samples under various conditions. The first analysis aimed at comparing the distribution of the means of the Spearman’s correlation of gene expression within the pathways extracted using MetPath and the distribution of the Spearman’s correlation of genes mapped within Kegg’s pathways. To retrieve genes involved in each KEGG pathway, we used Keggrest, an R package that provides a client interface for the KEGG REST server. We mapped the standard condition expression data obtained from the *E. coli* expression database mentioned above onto the extracted pathways, and we scored the average of the correlation for each pathway (Figure 2a). To further investigate the correlations of genes within the pathways, we repeated the analysis using the MetPath-extracted pathways, filtering the genes by their expression. We studied the gene correlations as a function of their expression by increasing the expression threshold and scoring the average of the Spearman’s correlations of gene expression within the pathways extracted (Figure 2b). To support the hypothesis that genes with closer reactions have a better correlation and, thus, smaller pathways offer a better starting point for analysis, we analyzed how the correlation is influenced by pathway lengths. We generated 15 sets of extracted pathways using three different conditions (standard, anaerobic, and tryptophan-supplemented) and different distance parameters ranging from 1 to 5. These sets were then used to score the average of genes involved in each pathway and their correlation. These values were used to perform linear regression (Figure 2c). The subsystems are annotations that link reactions to the pathways following the literature. In the model we used for *E. coli*, the subsystem annotations obtained from KEGG were used to perform a direct comparison of results. To score perturbations, for each pathway, we considered an aggregate perturbation score and the involved reactions. Then, a hit score was calculated for each subsystem by looking at how many reactions of the current pathway were associated with a subsystem. The hit scores were then multiplied by the aggregate perturbation score of the current pathway. This was performed for each extracted pathway, and then the values were collapsed by scoring the average for each subsystem. The KEGG analysis was performed by calculating the mean of the threshold value to select all genes with an up- or down-regulation equal to or above 65% in each condition. Up-regulated genes and down-regulated genes were compared to the KEGG pathways using KEGG Mapper.

### 2.6. Anaerobic Condition Analysis

To test MetPath functionalities we compared the expression data obtained under anaerobic conditions to data obtained under aerobic conditions. In both cases, we used the average values of three replicates from the expression data available in the literature [13]. We used the metabolic model iJO1366 from the BiGG database [14] for the *E. coli* strain K-12 MG1655. To constrain the metabolic model, the ATP production was set as the objective function and the M9 minimal medium was used as the culture medium. For the aerobic conditions, the growth rate was constrained to 0.4 h^−1^ [12] in order to activate the reactions involved in the production of the most important metabolites, and for the anaerobic conditions, the biomass production was decreased to 0.26 mmol/gDW/h and the uptake of O_2_ was decreased to −2 mmol/gDW/h.

### 2.7. Tryptophan Supplementation Analysis

To test the universal database, RNAseq data from a tryptophan-supplemented aerobic growth condition was used [7]. Since the universal database is based on array expression data, we could not perform a direct comparison of the expression values. Thus, we ranked the genes in the RNAseq data based on their expression. Then, we gave a value that was equal to the corresponding position in the ranked list. The same procedure was performed for the expression values of every condition within the database. These values were then used as the expression values. Then, the final aggregate perturbation score and the set of reactions that made up a pathway were calculated. The final aggregate perturbation scores were calculated as the average of the aggregate perturbation scores of a pathway across all the conditions of the database. To choose the set of reactions that made up a pathway, we considered those from the pathway with a higher aggregate perturbation score across all conditions and, thus, comprised the set of reactions that better represented and fitted the expression data. Using RegulonDB, we examined the relationship between the tna operon and the trpR transcription factor with the reactions within the extracted pathways in the tryptophan-supplemented condition. The tna operon is a well-known operon involved in tryptophan metabolism, which encompasses one regulator, tnaC, and two enzymes, tnaA and tnaB. TnaA, also known as Tryptophanase, catalyzes the cleavage of L-tryptophan to indole, pyruvate, and NH^4+^. TnaB is known as a transporter for tryptophan in *E. coli*. The trpR transcription factor negatively regulates the expression of the trp regulon in response to intracellular levels of tryptophan. For each pathway, we took into consideration the reactions in order to extract the involved genes; then, using RegulonDB, we analyzed which gene is related to the tna operon or to the trpR repressor.

### 2.8. Neurotransmitter Analysis

For each extracted neurotransmitter pathway from RECON1, we mapped the normalized expression values onto the genes of the reactions involved. The model was optimized for atp production, and the resulting value was used to constrain its own rate to obtain at least 90% of maximum atp production. Then, we added a demand reaction for each metabolite present in the biomass production formula, with an exception for cholesterol ester in the endoplasmic reticulum due to the inability of this model to produce this metabolite. We optimized each demand reaction one by one and divided the resulting scores by the total number of demand reactions added. These values were used to constrain the corresponding flux rate of the reactions from which the value was obtained. This step was not meant to obtain the exact production amount of the biomass components but to activate the upstream reactions needed for their synthesis. An additional constraint was added by forcing the model to produce a minimum amount of all studied neurotransmitters, with the aim of activating the upstream reactions needed for their production. Finally, to adapt the model to each neuron type, the production rates of the neurotransmitters expected to be associated with a specific neuron were maximized. We used MetPath to extract the neurotransmitters’ production pathways from every neuron and mapped the standardized gene expression onto reactions belonging to these pathways. Then, by summing these values within every pathway, we obtained a production score used to generate the heatmap. The results confirm that the algorithm has high reproducibility and specificity; moreover, the extracted paths are as good as those manually extracted using the literature or even better in some cases.

## 3. Results

### 3.1. Calculation of Condition-Specific Pathways for Production and Consumption of Metabolites

The MetPath computational workflow to analyze the change in metabolic production and degradation pathways in the network is as follows (Figure 1): To summarize the workflow, we first defined an estimated metabolic state based on the established metabolite uptakes and energy production estimates using flux balance analysis [15]. Calculation of an accurate and complete flux state is desired as incorrect or inactive reactions will lead to wrong or missing pathways, respectively. We then defined the production and degradation pathways for each metabolite in the network using the network structure and constraint-based pathway definition algorithms [15]. Briefly, for each separate metabolite, the local active network within a certain user-defined number of reactions upstream and downstream of the metabolite was extracted. Then, the flux state for this subnetwork was broken down into elementary modes, yielding pathways for the production and consumption of the metabolite as well as weightings for those pathways according to how much each mode contributes to metabolite production or consumption. While the flux solution in the subnetwork around the metabolite contains fluxes that do not involve the metabolite of interest, this elementary mode extraction serves to isolate only the pathways involved in the production or consumption of the metabolite of interest. While elementary mode calculation is not numerically tractable on the genome scale, it is efficient at defining pathways for small active subnetworks extracted around individual metabolites, thus bypassing the scaling issues typical of pathway calculation algorithms. The calculated elementary modes were then summed together to simultaneously represent all pathways involved in the production or consumption of the metabolite under this condition. The numerical values on each reaction are the flux contribution to production or consumption of the metabolite, which we scaled to a norm of 1 to serve as a relative weighting on expression fluxes.

Finally, using these defined pathways, we created an aggregate perturbation score for each production and degradation pathway based on the fold change of significantly changed metabolic genes within each pathway. Perturbed pathways thus represent network-integrated gene expression changes in the production or degradation potential of specific metabolites. The weights of the genes were assigned based on the flux carried by the reaction within the pathway for the metabolite of interest. Changes in genes that contribute larger flux reactions were weighted more heavily than genes whose reactions contribute little to the production or consumption of the metabolite of interest. Statistical *p*-values indicating the significance of the up-regulation or down-regulation of a pathway were calculated using nonparametric permutation tests.

In the initial validation of the biological significance of these pathways, we looked at gene knockout phenotypes. We obtained gene knockout data previously used to validate the *E. coli* metabolic model [16]. This dataset consists of the characterization of the Keio collection of genome-scale gene knockouts in different media, with corresponding growth rate effects of the knockouts. If genes were related within the pathways, it was expected that they would share a phenotype more often than randomly associated genes. We found that the variance in gene essentiality was 0.13 when compared to the MetPath pathways, which had an internal variance in the average gene essentiality of 0.087. Therefore, it appears that the MetPath pathways are substantially more similar in essentiality than all the genes in total, further supporting the relatedness of their genes. 

### 3.2. Definition of a Universal Pathway Database for E. coli Expression Analysis

As the definition of a metabolic state depends on the condition of interest, we defined a set of standard conditions that may be of interest to users and calculated the MetPath pathways for each of these conditions. We utilized the iJO1366 *E. coli* genome-scale metabolic network [16] and calculated the flux states for a representative set of 66 growth conditions, altering carbon and nitrogen sources as well as terminal electron acceptor. We combined the MetPath pathways for each metabolite in the network under these conditions into a single database by combining pathways that were shared between conditions (Matthews correlation coefficient between the pathways greater than 0.9) and leaving dissimilar pathways separate. The resulting pathway database consists of a set of condition-specific production and consumption pathways for each metabolite in the network. This database serves as a basis for pathway-based analysis of gene expression data across diverse conditions and is made available in the Supplementary Data File S1.

### 3.3. Comparison of MetPath Pathways to Manually Curated Pathways

To assess the ability of the MetPath *E. coli* pathway database in interpreting differential gene expression, we gathered 213 gene expression samples from *E. coli* K12 MG1655 grown under various conditions and genetic perturbations. We mapped this gene expression dataset onto the pathway database and examined the co-expression of genes within pathways. We calculated the correlation of genes within the same pathways compared to genes that do not share pathways (Figure 2a) and compared to pathways extracted from the KEGG database. We found that genes within the MetPath pathways are substantially more correlated. This correlation is dependent upon the pre-defined length of a MetPath pathway, with shorter distances associated with higher correlations. This indicates that the co-expression of genes along metabolic pathways tends to be highly colocalized. As the KEGG pathways tend to be significantly longer than the MetPath pathways, this colocalization bias leads to a lower total co-expression of genes in the KEGG pathways. Additionally, we found that more highly expressed genes tend to be more co-expressed within the same pathways (Figure 2b). This could indicate either a tighter gene expression regulation of highly expressed genes or a clearer correlation signal in highly expressed genes compared to low-expressed genes due to noises associated with measuring the latter. To explain the comparatively better performance of MetPath when compared to KEGG, we hypothesized that the shorter length of the MetPath pathways is beneficial. Indeed, we found that the correlation between genes in the MetPath pathways rapidly falls off as the distance between genes increases (Figure 2c), which is consistent with previous findings [17]. This indicates that the greater spatial localization of the MetPath pathways is beneficial for discovering locally co-regulated gene sets.

Looking at the TCA cycle as a case study, the KEGG pathway consists of 27 genes that map onto 19 reactions, including two genes, ybhJ and ydbK, that have no assigned functions in the metabolic model, and Ecocyc indicates their functions are putative or spurious. MetPath identifies 57 metabolites with production or consumption pathways that involve TCA cycle reactions. These pathways are generally shorter than the KEGG pathway, with a median length for the production pathways of six reactions, and a median length for the consumption pathways of five reactions. The pathway with the most reactions is the consumption pathway for FADH2, which involves 19 reactions on its own, many of which are minor variants of the same set of redox reactions. From observation, the MetPath pathways of the TCA cycle metabolites contain a large part of the canonical TCA cycle. For example, the production pathway for succinate contains the following reactions: ACONTa, ACONTb, AKGDH, CS, FRD2, FRD3, ICDHyr, NADH17pp, NADH18pp, and SUCOAS. The consumption pathway of succinate contains FUM, MDH, and SUCDi. Thus, between the production and consumption pathways for succinate, most of the canonical TCA cycle is represented. Notably, isozymes that are not used in the selected growth condition, for example, due to less energetically favorable stoichiometries, do not appear in the corresponding pathways.

### 3.4. MetPath Pathways Reveal Coordinated Expression Changes with Shifts in Environment 

**Figure 3:**
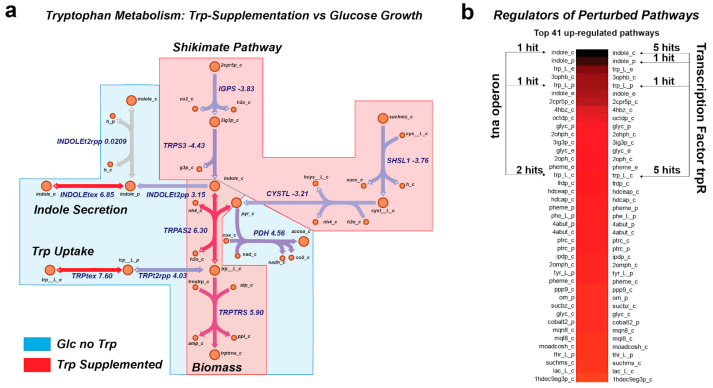
MetPath pathways highlight metabolic and regulatory shifts due to growth condition. (**a**) MetPath pathway scores under tryptophan supplementation reveal differential pathways of tryptophan production and consumption in a condition-specific manner, with red highlight indicating down-regulation and blue highlight indicating up-regulation. (**b**) Examination of highly perturbed metabolite production and consumption pathways reveals that many of the top pathways are regulated by trpR, a well-known regulator of tryptophan metabolism.

We were then interested in determining whether the MetPath pathways could identify functional differences between metabolic states based on gene expression data. By utilizing the available gene expression data for *E. coli* again, we examined two comparisons. First, we looked at the MetPath production and consumption pathways for tryptophan for *E. coli* grown aerobically on glucose with and without tryptophan supplementation (Figure 3). We found that due to the change in the underlying metabolic flux state calculated based on the flux balance analysis for each condition, the MetPath pathways differ substantially between the glucose-only case, where tryptophan must be synthesized de novo, and the tryptophan-supplemented case. This condition change is associated with both a clear expression change that is observed in the MetPath pathway scores as well as a known shift in the activity of the transcription factor regulating this pathway. An examination of highly perturbed metabolite production and consumption pathways reveals that many of the top pathways are regulated by trpR, a well-known regulator of tryptophan metabolism (Figure 3b).

**Figure 4:**
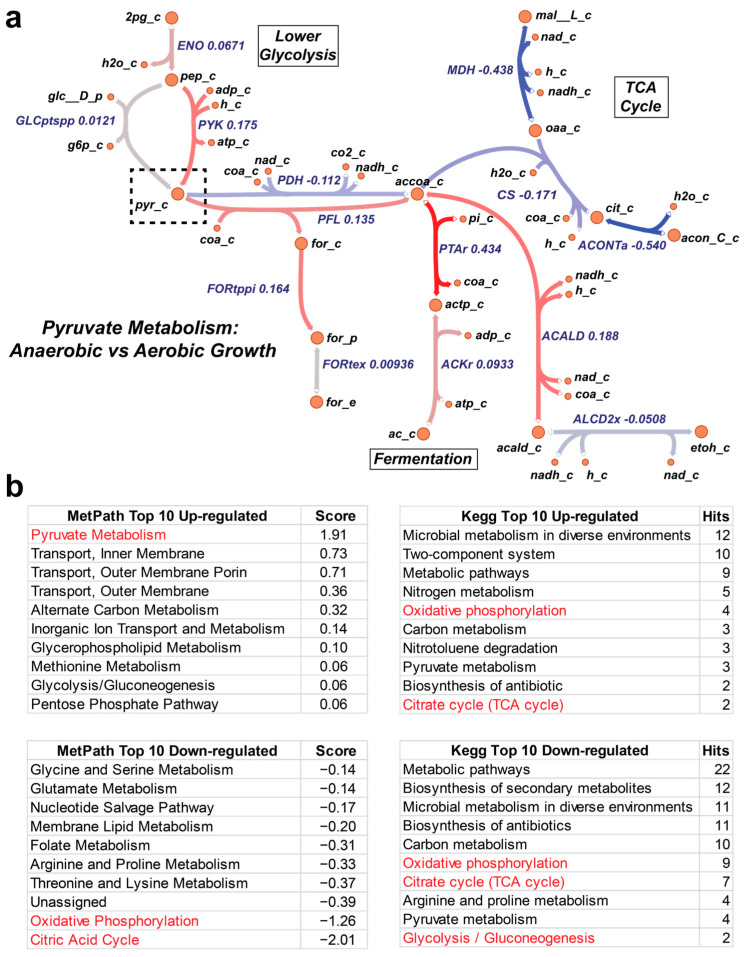
MetPath pathways resolve complex expression patterns. (**a**) Differential expression between aerobic growth and anaerobic growth of *E. coli* revealed by MetPath scores for pyruvate. (**b**) Examination of highly perturbed subsystems in MetPath and KEGG pathways shows that MetPath highlights pyruvate metabolism as being consistently up-regulated and oxidative phosphorylation and the citric acid cycle as being down-regulated, consistent with the expectations for an aerobic–anaerobic shift. Meanwhile, KEGG subsystems give conflicting results, with these pathways appearing in both up-regulated and down-regulated subsystems.

Second, we examined central energy and oxidative metabolism during aerobic-anaerobic shift in *E. coli*. Looking specifically at the pyruvate pathway, a key branch point in the oxidative/glycolytic shift (Figure 4), we once again observed a coordinated multi-gene response along the MetPath pathways. This indicates that a clear expression signature exists that can be mapped onto the metabolic network through the MetPath pathways to obtain an integrated signature with potentially greater statistical power than single gene-based expression analyses. An examination of highly perturbed subsystems in the MetPath and KEGG pathways shows that MetPath highlights pyruvate metabolism as being consistently up-regulated and oxidative phosphorylation and the citric acid cycle as down-regulated, which is consistent with the expectations for an aerobic–anaerobic shift. Meanwhile, the KEGG subsystems give conflicting results, with these pathways appearing in both up-regulated and down-regulated subsystems (Figure 4b). 

### 3.5. MetPath Pathways Recapitulate Canonical Cell Type-Specific Metabolic Functions

**Figure 5:**
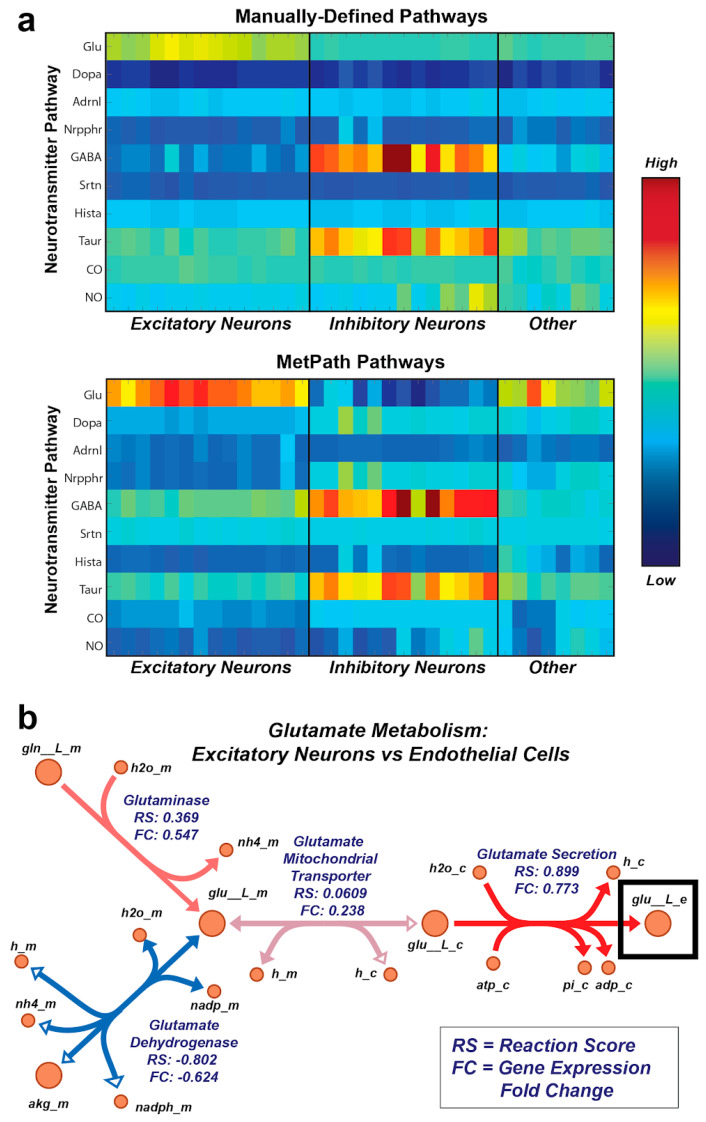
MetPath reveals cell-specific expression of neurotransmitter use in single-cell neural gene expression data. (**a**) Single-cell gene expression data mapped onto the metabolic pathways for neurotransmitter production for a representative set of neurotransmitters. Analysis of subtypes of neural cells revealed differential expressions of neurotransmitters that are consistent with canonical neurotransmitter use. (**b**) MetPath scores for glutamate production in excitatory neural cells compared to endothelial cells. A coordinated up-regulation of glutamate production and secretion genes and down-regulation of the primary glutamate degradation enzyme glutamate dehydrogenase was observed, consistent with the use of glutamate as an excitatory neurotransmitter.

Finally, we wanted to determine whether the MetPath pathways could identify functional metabolic differences across entirely different cell types using their gene expression alone. To this end, we utilized single-cell gene expression data from a set of 33 cell subtypes identified from human brain samples [18]. Given that the samples originated from the human brain, we were interested specifically in whether MetPath could identify differential use of metabolic pathways associated with neurotransmitters in these cell subtypes. We collected pathways for a representative set of neurotransmitters and mapped the expression data for the neural cell subtypes onto these pathways using the global human metabolic network reconstruction Recon 1 [19] (Figure 5a). Encouragingly, we observed clear differentiation of cell subtypes based on the expression of neurotransmitter pathways. These neurotransmitters matched their canonical use within particular neural cell subtypes, such as the association of GABA with inhibitory neurons and glutamate with excitatory neurons. Additionally, we identified unusual neurotransmitter use among particular subtypes of neurons, such as an up-regulation of the NO synthesis pathway among particular subtypes of inhibitory neurons. With the goal of comparing integrated pathway analysis with single-gene analysis, we again extracted the glutamate pathway as a case study (Figure 5b). We compared the expression of the glutamate pathway within excitatory neurons, where this pathway is canonically activated, and endothelial cells, where its role is unclear. As with *E. coli*, we observed that there is a coordinated multi-gene signature involving an up-regulation of glutamate production in the excitatory neurons. This signature includes an up-regulation of glutamate production and secretion genes and a down-regulation of the primary glutamate degradation enzyme glutamate dehydrogenase, which is consistent with the use of glutamate as an excitatory neurotransmitter. Thus, it appears that the integrated analysis using MetPath pathways reveals additional coordination that would be more difficult to see based on single-gene analysis.

## 4. Discussion

In this work, we developed a constraint-based modeling method, termed MetPath, to calculate metabolite production and consumption pathways for the purpose of differential analysis of gene expression data. We compared this method qualitatively and quantitatively to existing pathway databases, most notably KEGG, and found that the MetPath pathways showed higher intergene correlation within the same pathways across different conditions in *E. coli* K12 MG1655. We examined the performance of MetPath using two case studies in *E. coli*, looking at the tryptophan pathway during aerobic growth on glucose with and without tryptophan supplementation as well as glucose growth under a condition of aerobic–anaerobic shift. Finally, we looked at the ability of MetPath pathways to interpret cell-specific differences in gene expression, examining specifically neurotransmitter pathway expression in human neural cell subtypes based on single-cell transcriptomics data.

We examined the performance of MetPath pathways in comparison to KEGG pathways in predicting gene correlation, primarily with the goal of displaying the qualitative difference between the behavior of the pathways. This study was not intended to be a rigorous comparison of various pathway databases and pathway algorithms to determine the best performing set of pathways. Others have conducted such analyses [7], and sufficient increases in available validated data have not been made to warrant revisiting this effort. However, the results showing that the expression among genes was more correlated within the MetPath pathways than within the KEGG pathways across conditions lend credibility to the hypothesis that the localization of these pathways around the production and consumption of individual metabolites, as well as the condition-specific nature of the pathways, may yield some tangible benefits when performing pathway-based data analyses.

The basis for using a metabolic pathway to understand differential gene expression is rooted in the assumption that the expression difference is associated with a change in flux through the metabolic pathway. The link between gene expression and metabolic flux, which is the variable of greatest interest, is known to be indirect at best [20]. mRNA and enzyme levels are known to have only modest correlation [21], and the flux catalyzed per unit of enzyme depends also on metabolite levels, which can change between conditions [22]. Thus, rather than attempting to estimate differential flux levels directly using gene expression changes, we decided to ask the more addressable question of how gene expression changes have made the different ‘functions’ of the metabolic network more or less difficult based on the assumption that mRNA changes and enzyme changes are positively correlated [23].

To provide real case studies demonstrating the utility of MetPath pathways in understanding the functional significance of gene expression differences, we looked at the gene expression from *E. coli* grown under different conditions as well as the gene expression from different cell subtypes in the human brain. In each case, we found that highly perturbed pathways were directly tied to the functional difference between conditions or cell types. A similar result may be obtained by looking at the expression of individual genes in these cases. However, we observed a coordinated gene expression difference among several genes in the pathways in each case. Thus, statistical testing to examine this integrated gene expression change may yield additional power compared to non-pathway-based analysis methods.

This work fills a gap toward building simple and intuitive metabolic model-based statistical analysis of expression data. The benefits of the MetPath algorithm are that pathways are (1) automatically calculated, (2) intuitively defined, (3) condition specific, and (4) numerically tractable. We believe that this particular set of traits distinguishes the obtained pathways from existing pathway sets and enables the algorithm to be broadly useful across new data sets and organisms.

## Data Availability

The datasets supporting the conclusions of this article are included within the article and its additional files.

## Supplementary Materials

The following supporting information can be downloaded at https://www.mdpi.com/article/10.3390/metabo13111127/s1, Supplementary Data File S1.
